# Progress in Genito-Urinary Surgery

**Published:** 1904-04-23

**Authors:** 


### Progress in Genito-Urinary Surgery.

(*Concluded from page* 44.)

**Tuberculosis of the Urethra.**—A. L. Chute (Boston, Mass.)[12] has published an account of this very rare affection The patient, a married man of 35, for two years suffered from painful and frequent micturition, with occasional hæmaturia and slight urethral discharge. During the same period the left inguinal glands enlarged. The stream of urine became gradually smaller, and when first seen by Chute was very small and followed by dribbling. The urine contained a trace of albumin, a few shreds, and pus cells, cocci, and bacilli, but no gonococci. The urethra felt indurated. One and a half inch from the meatus was a hard bulbous enlargement, and at the penoscrotal junction a second one. Crepitus was yielded on moving the urethra laterally at the latter point, and a probe gave a grating sensation in the enlargements. The urethra was gradually dilated, and the micturition troubles relieved. With a forceps bits of calcified material were several times removed from the urethra. The glands in the left groin were excised, and showed amyloid infiltration, a rare condition, and usually due to tuberculosis in some part of the lymphatic area. The tubercle bacillus was not found. Chute has been unable to find any case recorded in which similar calcareous deposits were produced. Urethral tuberculosis affects men more than women, and may occur in childhood. In cases where a necropsy has been made other tubercular lesions of the genital or urinary tract have usually been present. There are two forms of lesion—granulations and ulcers.

**New Growths in the Tunica Vaginalis.**—Edward A. Balloch (Washington)[13] reports the case of a boy 16 years of age who had an enlargement in the upper part of the right scrotum which gradually increased in size for eight years. No cause was ascertainable. The scrotum reached nearly to the knees, and measured 21 inches in circumference at its largest point. Both testicles and cords felt normal. Three separate tumours were felt in the right side of the scrotum, and they were movable in relation to the cord, testis, and scrotal tissue. The diagnosis of fibroid growth in the tunica vaginalis was made, and operation showed that it was greatly enlarged, thickened, and gelatinous. The upper tumour had a pedicle attaching it in the inguinal canal, the other two showed no distinct attachments. These tumours, the tunica vaginalis, and accidentally the testicle were removed. The tumours were soft fibromata, with areas of fatty and myxomatous degeneration. Only one case at all resembling this was found recorded. Minute fibrous growths in hydroceles, following treatment by iodine injections, have been several times recorded.

**Operations on the Testicles and Vasa Deferentia.**—Pascal[14] has experimented on animals, and shown that it is possible to unite the two testicles and that a single vas deferens will suffice for both. Thus the epididymis can be removed for tuberculosis, and the testis retained and a natural channel be left for the issue of its secretions. After resecting the epididymis or vas on the affected side the rete testis is opened and examined, and if found unaffected, is implanted on the sound testicle. This is easily done after removing the inter-testicular septum. Four cases have been operated upon in this way. The functional activity of the implanted testicle is said to be maintained. The procedure is named "synorchidia." H. Vulliet (Lausanne)[15] discusses the question of the permeability of a vas deferens which has been sutured after division. So far as the author knows, although the divided vas has been sutured, there is lacking both anatomical and functional proof that a successful result has been achieved. The question is one of great importance, for the vas is liable to be unintentionally divided, *e.g.* in difficult hernia operations, or intentionally when a diseased portion is resected. After complete or partial resection of the epididymis, it is desirable to join the vas either to the stump of the epididymis, or to the testicle itself, if this can be successfully done. The author then states that he is able to give anatomical proof that a sutured vas deferens is capable of complete recovery in dogs and therefore probably in man. The suture material has to be very fine. The diameter of the vas is not more than 2 mm. and its lumen not more than $\frac{1}{2}$ mm. The thickness of the wall permits of suturing without penetrating the lumen. In four sutured vasa deferentia the author found, by serial sections made subsequently and examined under the microscope, that three were permeable and lined with normal epithelium. A drawing is given of the microscopical appearances of a transverse section of the sutured portion six months after the operation. J. Lynn Thomas[16] describes a method he adopted in suturing a divided vas deferens. The patient had had one vas deferens completely divided, and the other partially, by his wife in a fit of jealousy. The division had been made with a surgical knife when the man was intoxicated. On the side where the vas was completely divided there was a hard swelling the size of a pea, one inch above the testicle. This turned out to be a collection of semen in a fascial sheath between the divided ends of the vas deferens. The testicular end of the vas was cut obliquely with a cataract knife. The urethral end was split up longitudinally for one inch on one side and for half an inch on the opposite side, thus leaving two tails. The free end of the testicular portion was sutured with its lumen applied to the lumen of the other part, with fine silk sutures, and the tails enveloped round the testicular portion to counteract the disruptive force of the weight of the testicle. Layers of fascia were also wrapped round the sutured part. Nine months later there was nothing abnormal as regards the testicle, and the thickening of the vas was about the same size as at the conclusion of the operation. The patient considered the operation had been a perfect success and his wife was four months pregnant. Obviously the value of the case as proof of permeability of the sutured vas is invalidated by the fact that the condition of the other vas is not known. William Thorburn[17] describes a modified operation of castration. The object of the modification is to secure more aseptic results. In the ordinary operations, partly owing to the skin of the scrotum being difficult to disinfect, partly to the common existence in cases requiring castration, of septic sinuses or ulcers due to tubercular or malignant disease, the wound is very liable to become septic. The operation described by the author gives high access to the cord, closes securely the abdominal walls and avoids the danger of suppuration in the inguinal region. These results are achieved by at first only exposing and operating in the inguinal region. A 1½ inch incision there is deepened through the external oblique until the spermatic cord is exposed. That structure is then traced well down into the pelvis, and there clamped, and ligatured above the clamp after transfixation. Another ligature is placed ½ inch below the clamp, and the cord cut across close to the distal side of the instrument. The internal oblique is then sutured to the deep layer of Poupart's ligament, and the external oblique sutured and the skin wound closed. Then the scrotum is for the first time uncovered, and the testicle and cord quickly removed through a separate incision. The operation has been found to successfully accomplish the objects intended, for in all cases the upper wound has healed *per primam*, although in two cases the lower wound became infected.

[12] (Abst.) Lancet, Nov. 7, 1903, p. 1315. [13] Med. Rec., Jan. 9 1904, p. 75. [14] (Abst.) Clin. Jour., Oct. 7, 1903, p. 400 [15] Zentralb. f. Chir., No. 2., Jan. 16, 1904, p. 36. [16] Brit. Med. Jour., Jan. 2, 1904, p. 13. [17] Ibid., Jan. 16, 1904, p. 127.

